# Influence of tropical upright pasture structural and chemical characteristics on lamb grazing time

**DOI:** 10.1371/journal.pone.0242642

**Published:** 2021-04-15

**Authors:** Jalise Fabíola Tontini, Cesar Henrique Espírito Candal Poli, Viviane da Silva Hampel, Mariana de Souza Farias, Neuza Maria Fajardo, Joseane Anjos da Silva, Luis Henrique Ebling Farinatti, James Pierre Muir

**Affiliations:** 1 Department of Animal Science, Universidade Federal do Rio Grande do Sul, Porto Alegre, Rio Grande do Sul, Brazil; 2 Department of Animal Science, Universidade Federal do Acre, Cruzeiro do Sul, Acre, Brazil; 3 Texas A&M AgriLife Research, Texas A&M University, Stephenville, Texas, United States of America; Universidade Federal de Viçosa, BRAZIL

## Abstract

Tropical pasture canopy characteristics can alter lamb ingestive behavior. Our study evaluated the ingestive behavior of young lambs in different tropical pastures to identify which variables interfere in their grazing activity. Two years of study were carried out with 54 weaned lambs distributed in three different pasture canopies: 1) monoculture of an upright grass, guinea grass (*Panicum maximum*; GG); 2) monoculture of a shrubby legume pigeon pea (*Cajanus cajan*; PP) and 3) contiguous paddock with half GG and half PP (GP). The experiment was set out in a randomized complete block design (3 blocks). Lamb ingestive behavior was observed from sunrise to sunset with records every 5 minutes. To identify the main variables that affected lamb grazing activity, a multivariate analysis of the Decision Tree was performed. Our results showed that there was no difference in the ingestive behavior parameters of young lambs in different canopies (*P* > 0.05). There was interaction among the canopies and the experimental periods for the variables idleness time and biting rate (*P* ≤ 0.05). Lambs in all canopies showed more idleness time in the first evaluation period. Lambs in canopies containing grass (GG and GP) exhibited greater bites per minute throughout the experimental period. Lamb grazing time increased 40% as experimental period progressed and plants matured. The Decision Tree identified leaf:stem ratio as the variable that most influenced lamb grazing time in GG and GP canopies while in the PP, grazing time was directly related to canopy height. The behavior of young lambs on tropical pasture is variable as there is a change in the behavioral response to canopy characteristics over time. In addition, the grazing time of these animals can be estimated by means of variables related to canopy structural characteristics (leaf:stem ratio and height) together with chemical variables.

## Introduction

Knowledge of the plant-animal interface becomes an indispensable factor when working with pasture production systems. In pastoral ecosystems, herbivores develop grazing mechanisms or tools that make up what is called ingestive behavior [1]. Based on the existing literature, there are two main lines of research on ingestive behavior, one that explores animal response under different environmental conditions, with responses based on conditions extrinsic to animals [2–5], and the second that studies behavioral responses related directly to the different physiological conditions of animals, making a direct relationship between behavior and their intrinsic characteristics [6–8].

From an extrinsic perspective, ingestive behavior may be one response, among other factors, to the type of pasture offered to the animal. In temperate pastures these behavioral responses are already well elucidated. There are conceptual models structured from scientific studies showing that pasture structure has an important effect on herbivore ingestive behavior characteristics and is directly responsible for the nutrient quantity ingested by grazing animals [3, 9–13]. However, studies of lamb grazing behavior on tropical pastures are scarce, and the existing results are inconclusive. In addition to the diverse growth habits and morphology of tropical forage species, there is structural variability over time.

In regions with tropical and subtropical climate, pastures are widely used in beef cattle production systems and appear to be expanding for sheep production. Despite this, the production of lambs on pastures with high productivity is challenging and some studies indicate unsatisfactory performance of the animals, below 100 g liveweight gain/day [14–16]. The productivity of lambs on pasture is attributed to their ability to harvest nutrients efficiently and effectively from the pasture [17], and the understanding of ingestive behavior is an important tool for directing management practices to obtain better animal performance. Therefore, before making adjustments to tropical pasture management to improve animal performance, it is essential to understand the dynamics of ingestive behavior of these animals.

In addition to the type of forage, the intrinsic aspects of the animals can also interfere in the ingestive behavior. Different physiological conditions of the animals require different nutritional inputs. In tropical grazing systems, sheep identify the heterogeneity of the physical and nutritional characteristics of plant components to meet their nutritional requirements. Diet selectivity is an animal strategy to avoid nutritional deficiencies and adaptive behavior allows grazing animals to perceive and interpret certain circumstances and thus modify their behavioral responses [18]. Assessing the behavior of grazing lambs on a fast-growing pasture is extremely important. In addition to high nutritional demand, body size is associated with the time and energy spent selective grazing. The digestive capacity and body weight of these young animals are related to the choice of forage. This is one of the reasons why they have small mouths with the ability to select parts of a plant, generally resulting in a nutritional quality superior to the average offered in the pasture as a whole [19]. Therefore, to generate sustainable sheep production systems in tropical and subtropical environments, it is important to understand the factors that affect grazing lamb ingestive behavior [20, 21]. There is a lack of studies that show how lambs, which are highly selective, have high feed quality requirements compared to adults, and possess small mouths, graze upright tropical pastures with variable structure and nutritive value.

Many studies have already been carried out with the objective of understanding the ingestive behavior of animals grazing different species of forage [22, 23]. However, few studies have characterized the ingestive behavior of young lambs in different upright tropical pastures with the presence of grasses and/or legumes. Given the above, this work is a sequence of research already carried out by the same group of studies [14, 16, 20], the objective of this work was to evaluate the ingestive behavior of lambs under conditions of continuous grazing submitted to different upright tropical species canopy and to identify which variables influence the grazing activity of these animals.

## Material and methods

This study was approved by the Ethics Committee on Animal Use [Comissão de Ética no Uso de Animais da Universidade Federal do Rio Grande do Sul (CEUA-UFRGS)–project n° 27830] and conducted at the Research Station of the Universidade Federal do Rio Grande do Sul, Eldorado do Sul, Rio Grande do Sul State, Brazil (30°05’11" S, 51°39’09" W), 46 m above sea level. The climate is subtropical humid ‘cfa’ according to the Köppen (1948) classification. The cfa classification is characterized by hot summers with temperature averages over 22°C in the hottest month and rains distributed evenly over the year [24].

### Animals, treatments and experimental design

The experiment was repeated for 2 years (from 1 February to 25 April, 2015 and from 10 January to 12 April, 2016). The pasture and animal variables were evaluated every 21 days. The experimental area was 1.8 ha. Fifty-four castrated male lambs were used as testers, aged 3–4 months, weighing on average 22.6 ± 0.265 kg in Year 1 and 20.4 ± 0.310 kg of live weight (LW) in Year 2. Each year, 6 lambs were allocated per paddock, nine paddocks of 0.2 ha each were established, and these were considered experimental units. The animals were distributed by weight and fecal egg count (FEC), so that all paddocks had similar weight and total parasitic contamination of the animals. The animals were assigned to different tropical upright grass and legume canopies: 1) monoculture of guinea grass (*Panicum maximum* cv. IZ-5; GG); 2) monoculture of pigeon pea (*Cajanus cajan* cv. anão; PP) and 3) contiguous areas with half the paddock with GG and half with PP (GP). The area received fertilizer (200 kg N-P_2_O_5_-K_2_O/ha; 5-20-20) and lime (5 mg/ha) prior to planting and the soil is classified as Typic Paleudul (US taxonomy) or sandy clay loam Acrisol (FAO classification). The guinea grass areas also received chemical fertilizer, with application of 150 kg of urea/ha one month before the animals entered the paddocks. The paddocks were arranged in a randomized block design (3 blocks). In order to evaluate lamb performance, they were weighed every 21 days, with prior fasting of liquids and solids for 12 hours.

### Canopy assessments

The lambs remained on continuous grazing. To estimate the forage mass, every 21 days six samples of 0.25 m^2^ were cut close to the ground in each paddock; three of these represented the average height of the paddock and three were collected at random. Sub-samples of approximately 100 g were taken from each sample to carry out structural separation into leaf and stem plus sheath. Forage allowance was calculated using the methodology described by Sollenberger et al. [25] (Forage allowance = forage mass (kg/ha) / animal live weight (kg/ha)). The adjustment of stocking rate was carried out every 21 days using the “put-and-take” technique [26], so that the treatments presented a supply of similar mass per kg LW. The stocking rate is expressed in animal unit (AU), which corresponds to an animal of 450 kg LW (1 AU = 450 kg LW). The average real forage allowance of treatments was 3.4 kg forage mass/kg LW). According to “put-and-take” technique, there were two groups of animals, one called “testers”, represented by the animals that remain throughout the experimental period in grazing and express the effects of treatments, and the other group called “regulators”, used only to control the growth of the pasture and keep the herbage allowance at the desired level.

Canopy height was obtained every 21 days. Fifty points were randomly measured in each paddock [27]. In GP paddocks 25 points were measured in the guinea grass area and another half in the pigeon pea area.

To evaluate the nutritional values of each feeding system, samples were collected every 21 days by grazing simulation termed “hand plucking” technique [28]. These samples were dried in a forced-air oven at 55°C until constant weight and ground through a 2-mm sieve. This material was used to determine DM (method n° 930.15), organic matter and mineral matter (OM and MM, method n° 942.05) and crude protein (CP, method n° 984.13) according to the Association of Official Analytical Chemists [29], ether extract (EE) [30], neutral detergent fiber (NDF) [31] and acid detergent fiber (ADF) [32]. Total digestible nutrients (TDN) were obtained by the equation described by Sniffen et al. [33].

### Ingestive behavior

Three evaluations of animal daytime ingestive behavior were performed [34]. The animals were observed from sunrise to sunset, recording every 5 minutes whether lambs were grazing, ruminating or idling. In the interval between observations, biting rate was recorded for 20-bite time [35]. Within each paddock, each animal had a different colored necklaces to facilitate identification.

### Statistical analysis

Analysis of variance was performed with repeated measures over time to determine the effects of treatments on canopy and animal variables using the MIXED procedure of the statistical program SAS (version 9.4—SAS Institute Inc., Cary, NC, USA). Treatment, block (within each year) and period were considered as fixed effects. Year and Treatment*block (within each year) interaction were considered as random effects.

The data were submitted to the Shapiro-Wilk normality test. Exponential transformation was performed on lamb average daily gain (ADG) data. Correlations among the variables were evaluated using Spearman correlation analysis.

The multivariate Decision Tree analysis was performed by JMP software (version 12—SAS Institute Inc., Cary, NC, USA). This analysis indicates which factors most affected animal response to grazing time. Independent variables included lamb initial body weight and biting rate as well as canopy height, leaf:stem ratio, forage allowance, OM, CP, EE, mineral matter, NDF, ADF and TDN.

## Results

### Canopy characteristics

Forage harvested by the animals in the different pasture canopies showed adequate nutritional value, with CP values above 15% of DM and TDN close to 60% of DM. There was an interaction between canopy type and period for all forage nutritive value variables (Table 1). The canopy*period interaction for CP levels (*P* = 0.024) showed that GG had the lowest CP values, mainly in Periods 2 and 3. PP had the highest CP values, but it did not differ from the values found in the GP, independent of the evaluated period. The highest values of EE (*P* = 0.0412) were found in PP and GP canopies, mainly in the first and second period of the experiment. The values of mineral matter showed a canopy*period (*P* < 0.0001) interaction. PP presented the lowest values in the last evaluation period, while GG herbage presented the highest mineral matter content in the final period of the study. The fiber content of the pasture (NDF and ADF; *P* < 0.0001) was higher in GG and GP canopies compared to PP and were even higher in the last evaluation periods. The TDN (*P* < 0.0001) interaction showed that, in canopies with the presence of the grass (GG and GP), the amount of TDN decreased over the experimental periods as plants matured. In PP, the decrease occurred only from the first to the second period, which shows that the legume can recover or maintain its nutritional value with the advance of production cycle.

**Table 1:** Qualitative characteristics of different tropical pastures available to grazing young lambs in southern Brazil.

Forage nutritive value (%/kg of dry matter)	Per.	Canopy*			Period average	*P* value		
		Guinea grass (GG)	Pigeon pea (PP)	GG + PP		Canopy	Period	Canopy* Per.
Organic matter	I	90.6 ± 1.06 bc	90.7 ± 1.04 bc	90.7 ± 1.09 cd	90.7 ± 0.61	0.0052	0.5620	<0.0001
	II	88.6 ± 1.03 e	90.6 ± 1.04 bcd	89.8 ± 0.96 cde	89.7 ± 0.58			
	III	87.9 ± 0.94 de	92.4 ± 0.75 a	89.95 ± 0.75 c	90.1 ± 0.50			
Crude protein	I	15.7 ± 0.49 bc	19.7 ± 0.57 a	19.5 ± 0.64 ab	18.3 ± 0.37	0.0019	0.8305	0.0240
	II	15.5 ± 0.58 c	21.0 ± 0.65 a	19.0 ± 0.50 abc	18.5 ± 0.40			
	III	15.2 ± 0.57 c	21.6 ± 0.81 a	18.3 ± 0.57 abc	18.4 ± 0.46			
Ether extract	I	3.6 ± 0.13 ab	4.1 ± 0.30 a	4.07 ± 0.18 abc	3.9 ± 0.12 A	0.0472	<0.0001	0.0412
	II	2.8 ± 0.18 cd	4.2 ± 0.35 a	4.0 ± 0.20 abc	3.7 ± 0.16 A			
	III	2.4 ± 0.06 d	3.1 ± 0.11 bcd	3.4 ± 0.23 abcd	3.0 ± 0.10 B			
Mineral matter	I	9.4 ± 0.14 cd	9.3 ± 0.15 cd	9.3 ± 0.18 d	9.3 ± 0.09 C	0.0001	<0.0001	<0.0001
	II	11.4 ± 0.19 b	9.4 ± 0.15 cd	10.2 ± 0.18 c	10.3 ± 0.13 A			
	III	12.1 ± 0.17 a	7.6 ± 0.16 e	10.05 ± 0.19 c	9.9 ± 0.21 B			
Neutral detergent fiber	I	64.9 ± 0.39 a	45.7 ± 0.88 e	51.2 ± 0.28 cde	54.0 ± 0.85 B	<0.0001	<0.0001	<0.0001
	II	62.2 ± 0.47 ab	48.8 ± 1.38 de	54.3 ± 0.82 cd	55.1 ± 0.77 B			
	III	65.1 ± 0.27 a	56.4 ± 1.37 bc	62.4 ± 0.81 ab	61.3 ± 0.64 A			
Acid detergent fiber	I	32.1 ± 0.45 bc	27.7 ± 0.48 d	29.7 ± 0.39 cd	29.9 ± 0.30 B	0.0042	<0.0001	<0.0001
	II	37.3 ± 1.08 a	30.4 ± 0.71 c	34.4 ± 0.50 ab	34.1 ± 0.53 A			
	III	34.2 ± 0.57 b	34.9 ± 1.03 ab	34.9 ± 0.76 ab	34.7 ± 0.46 A			
Total digestible nutrients	I	63.2 ± 1.14 ab	67.8 ± 0.95 a	67.2 ± 1.32 a	66.1 ± 0.69 A	0.0982	<0.0001	<0.0001
	II	59.8 ± 0.98 bc	58.2 ± 1.39 bc	57.8 ± 1.20 bc	58.6 ± 0.69 C			
	III	58.9 ± 0.70 c	66.4 ± 1.50 a	57.0 ± 1.13 c	60.7 ± 0.77 B			

The means and their respective standard errors are presented.

^a,b^ Means in the same forage nutritive value followed by different lowercase differ significantly in the interaction canopy*period (*P* ≤ 0.05);

^A,B^ Means in the same forage nutritive value followed by different capital letter differ significantly among periods (*P* ≤ 0.05);

*Canopy: Guinea grass = monoculture of guinea grass (*Panicum maximum* cv. IZ-5); Pigeon pea = monoculture of pigeon pea (*Cajanus cajan* cv. Anão); GG + PP = 50% GG and 50% PP. Per.: period.

Forage mass data showed that the different canopies averaged >4000 kg DM/ha (Table 2). Leaf:stem ratio (Table 2) showed no difference among canopies with a mean of 0.51 (*P* = 0.1046). There were differences in leaf:stem ratio among periods, with a decrease over time (*P* < 0.0001). Canopy height was negatively correlated with leaf:stem ratio in PP (r = -0.60, *P* < 0.0001). Canopy height showed an interaction with period (*P* < 0.0001, Table 2). The tallest height (134 ± 3.3 cm) was observed in the third period in PP, differing from other periods and canopies (GG and GP). However, PP height in the initial and intermediate period of the study (103 ± 8.0 and 110 ± 6.9 cm, respectively) was not different from the height of GP (78 ± 2.4 cm), but it differed from the height found in GG (44 ± 1.5 cm). GP did not differ from GG, and the height of GG was different (*P* < 0.0001) among the different periods. The proportion of leaf blade in relation to the forage mass was similar among canopies. In the GG canopy, leaf mass represented 24.2% of the forage mass with an average of 1647.8 kg DM/ha; in the PP it represented 25.4% (1429.7 kg DM/ha); and for the GP a percentage of 26.3% (1347.3 kg DM / ha). Forage allowance was also similar among canopies, with 3.6 kg forage mass/kg LW for GG with stocking rate of 1919 kg LW/ha (4.3 AU/ha); for PP showed 3.5 kg forage mass/kg LW and stocking rate of 1611 kg LW/ha (3.6 AU/ha); and 3.0 kg forage/kg LW for GP with stocking rate of 1708 kg LW/ha (3.8 AU/ha).

**Table 2:** Leaf: Stem ratio, forage mass and canopy height of different tropical pastures available to grazing lambs in southern Brazil at different experimental periods.

Per.	Canopy*			Period average	*P* value		
	Guinea grass (GG)	Pigeon pea (PP)	GG + PP		Canopy	Period	Canopy*Per.
	*Leaf*: *Stem ratio*						
I	0.61 ± 0.023	0.50 ± 0.026	0.58 ± 0.026	0.56 ± 0.022 A	0.1046	<0.0001	0.0781
II	0.54 ± 0.026	0.38 ± 0.019	0.50 ± 0.010	0.47 ± 0.022 B			
III	0.48 ± 0.013	0.46 ± 0.013	0.44 ± 0.003	0.41 ± 0.025 C			
	*Forage mass (kg DM/ha)*						
I	5314 ± 446.4	4033 ± 123.6	4218 ± 165.1	4521 ± 348.8 B	0.2901	0.0354	0.0984
II	7737 ± 491.9	6588 ± 647.6	5894 ± 504.4	6740 ± 564.0 A			
III	7393 ± 449.8	6295 ± 809.1	5264 ± 405.3	6317 ± 671.8 A			
	*Canopy height (cm)*						
I	46 ± 2.1 de	103 ± 8.0 bc	84 ± 6.3 bcd	78 ± 4.1 AB	0.0027	<0.0001	<0.0001
II	41 ± 2.4 de	110 ± 6.9 bc	73 ± 2.7 ce	75 ± 3.8 B			
III	46 ± 3.2 de	134 ± 3.3 a	77 ± 2.5 bcd	86 ± 4.0 A			

The means and their respective standard errors are presented.

^A,B^ Means followed by different capital letter differ between periods (*P* ≤ 0.05);

*Canopy: Guinea grass = monoculture of guinea grass (*Panicum maximum* cv. IZ-5); Pigeon pea = monoculture of pigeon pea (*Cajanus cajan* cv. Anão); GG + PP = 50% of the paddock with guinea grass and 50% with pigeon pea. Per.: period.

### Animal performance and ingestive behavior

Lamb ADG showed an interaction between canopy and period (*P* < 0.0001), demonstrating that animals in GG and GP had greater capacity to maintain a constant ADG throughout the experimental periods than those grazing solely PP. The animals that grazed only legume (PP) had weight losses in the second period. Despite this interaction, there was no difference among treatments (*P* = 0.40), with mean values of 71 ± 8 for GG, 54 ± 9 for PP and 64 ± 8 g/day for GP. Likewise, there was no difference for weight gain by area, with an average of 18.6 ± 7.46 kg/ha/day (*P* = 0.25). There was a correlation between leaf:stem ratio and ADG in PP (r = 0.42, *P* < 0.0001).

All variables of ingestive behavior were not influenced by canopy type, but by period (*P* < 0.0001). The variations over time were important either independently or interacting with canopy type, as can be observed in Table 3. There was an interaction between canopy*period for idling time (*P* = 0.0390) and biting rate (*P* = 0.001). Idling time indicated that the longest times were recorded in the first period for all canopies. For the biting rate variable, this interaction showed that, in the canopies with the presence of grass (GG and GP), there was an increase in the number of bites per minute from the beginning to the end of the experiment. These values did not differ from the PP canopy containing only the legume; however, in this case the biting rate did not change over the experimental periods.

**Table 3:** Means of ingestive behavior of lambs on tropical pasture in three different evaluation periods.

Variables	Per.	Canopy*			Period average	*P* value		
		Guinea grass (GG)	Pigeon pea (PP)	GG + PP		Canopy	Period	Canopy*Per.
Grazing (min.)	I	360.4 ± 9.04	339.3 ± 10.08	367.4 ± 17.04	356.0 ± 7.35 C	0.6100	<0.0001	0.1964
	II	450.9 ± 7.93	437.6 ± 10.44	455.5 ± 11.90	448.1 ± 5.91 B			
	III	484.4 ± 19.59	499.8 ± 9.94	492.4 ± 15.18	492.2 ± 8.83 A			
Idleness (min.)	I	248.7 ± 9.84 a	251.8 ± 8.15 a	226.9 ± 15.63 a	242.2 ± 6.80 A	0.3287	<0.0001	0.0390
	II	126.2 ± 10.27 b	148.2 ± 10.22 b	122.3 ± 11.27 b	132.2 ± 6.17 B			
	III	131.76 ± 20.39 b	139.6 ± 7.98 b	135.4 ± 12.67 b	135.6 ± 8.34 B			
Rumination (min.)	I	110.9 ± 6.37	129.0 ± 9.36	125.7 ± 9.02	121.8 ± 4.84 B	0.8814	<0.0001	0.2163
	II	143.2 ± 7.63	134.6 ± 6.32	132.3 ± 5.67	136.7 ± 3.79 A			
	III	103.5 ± 6.55	77.6 ± 7.94	92.1 ± 7.09	91.1 ± 4.25 C			
Bite rate (per min)	I	20.1 ± 1.97 c	25.3 ± 1.24 abc	22.9 ± 1.53 c	26.1 ± 0.92 C	0.9087	<0.0001	0.001
	II	27.6 ± 0.98 ab	26.5 ± 1.60 abc	25.7 ± 0.98 ab	26.6 ± 0.70 B			
	III	30.0 ± 0.95 ab	25.8 ± 1.20 abc	30.4 ± 1.20 a	28.8 ± 0.67 A			

The means and their respective standard errors are presented.

^a,b^ Means in the same variable followed by different lowercase differ significantly in the interaction canopy*period (*P* ≤ 0.05);

^A,B^ Means in the same ingestive behavior variable followed by different capital letter differ significantly between periods (*P* ≤ 0.05);

*Canopy: Guinea grass = monoculture of guinea grass (*Panicum maximum* cv. IZ-5); Pigeon pea = monoculture of pigeon pea (*Cajanus cajan* cv. Anão); GG + PP = 50% of the paddock with guinea grass and 50% with pigeon pea. Per.: period.

The grazing and ruminating times underwent changes in the different evaluation periods (Table 3). On average, there was an increase in the grazing time of approximately 40% throughout the experiment. The rumination time recorded in the second period was longer than the other periods. A positive correlation existed between the fiber content, represented by ADF, with grazing time (r = 0.55, *P* < 0.0001).

### Decision Tree analysis

Decision Tree analysis was performed with the grazing time data to understand which variables (already mentioned in the section “Statistical analysis”) influenced lamb grazing time in each canopy. According to the analysis, in the GG paddocks, leaf:stem ratio and percentage of ADF of the forage explain 65% (R^2^ = 0.654) of the model, being the variables of greatest influence in animal grazing time. In PP paddocks, canopy height along with TDN and ADF of the forage explained 57% (R^2^ = 0.571) of the model for grazing time. In GP canopy, the percentage of ADF and leaf:stem ratio explained 65% (R^2^ = 0.649) of the grazing time observed.

**Fig 1:**
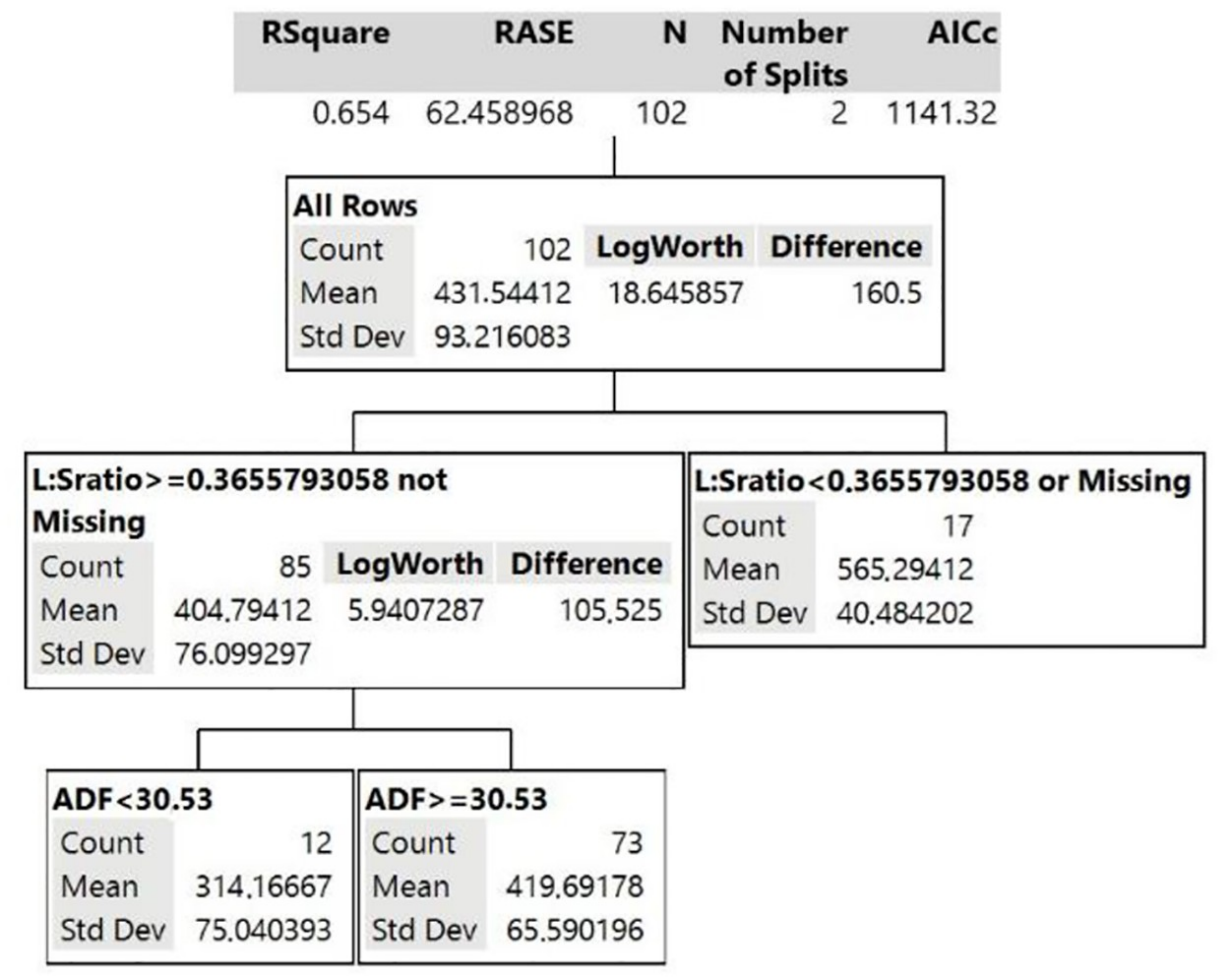
Decision Tree model for grazing time behavior variable of the animal grazing guinea grass (GG canopy).

As observed in Fig 1, the Decision Tree model for GG showed that the variable with the greatest interference in grazing time was leaf:stem ratio in the first division of the decision tree, explaining 42% of the model. Therefore, this analysis estimates that in pastures with leaf:stem ratio greater than 0.37, the average daily grazing time of the animals will be 404.8 minutes. If the leaf:stem ratio is less than 0.37, the grazing time of the animals will be greater, with estimated average of 565.3 minutes/day. In the second division of the Decision Tree model for GG, considering only the canopy with leaf:stem ratio greater or equal to 0.37, ADF content appears as the second variable with the greatest influence on grazing time. It estimates that if GG has ADF >30.5% of DM, grazing time will be approximately 420 minutes/day. If this ADF is lower than 30.5%, grazing time decreases to 314 minutes/day.

**Fig 2:**
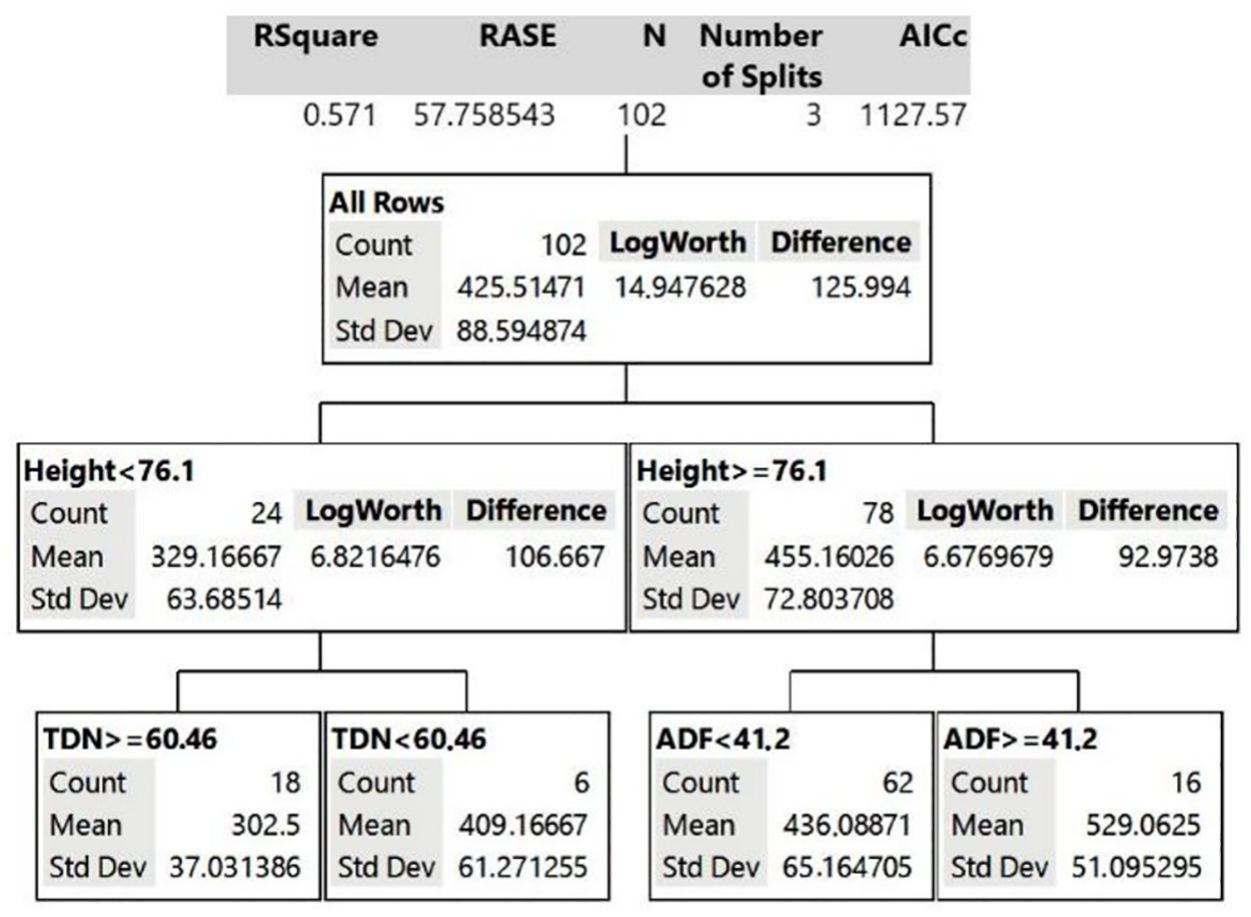
Decision Tree model for grazing time behavior variable of the animal grazing pigeon pea legume (PP canopy).

The Decision Tree model for grazing time in PP differed from GG (Fig 2). The main variable affecting lamb grazing time in PP was not related to the nutritional quality of the canopy but, rather, its structure. The PP height contributed to 37% in the Decision Tree model. The model estimated that if PP height is <76 cm, grazing time will be on average 329 minutes/day; however, if PP height is ≥76 cm, lamb grazing time will increase to 455 minutes/day. In the second division of the Decision Tree for PP, when height <76 cm, the percentage of TDN was the second variable that most affected grazing time. If TDN is greater than 60%, the estimated grazing time is 302.5 minutes/day. However, if that same pasture has a TDN <60%, the grazing time increases to 409 minutes/day. On the other hand, when PP is taller than 76 cm, ADF is an important determinant of grazing time. Therefore, in taller PP canopy with ADF lower than 41%, lambs graze for 436 minutes/day, but when this same pasture has ADF >41%, the estimated grazing time is longer, 529 minutes/day.

**Fig 3:**
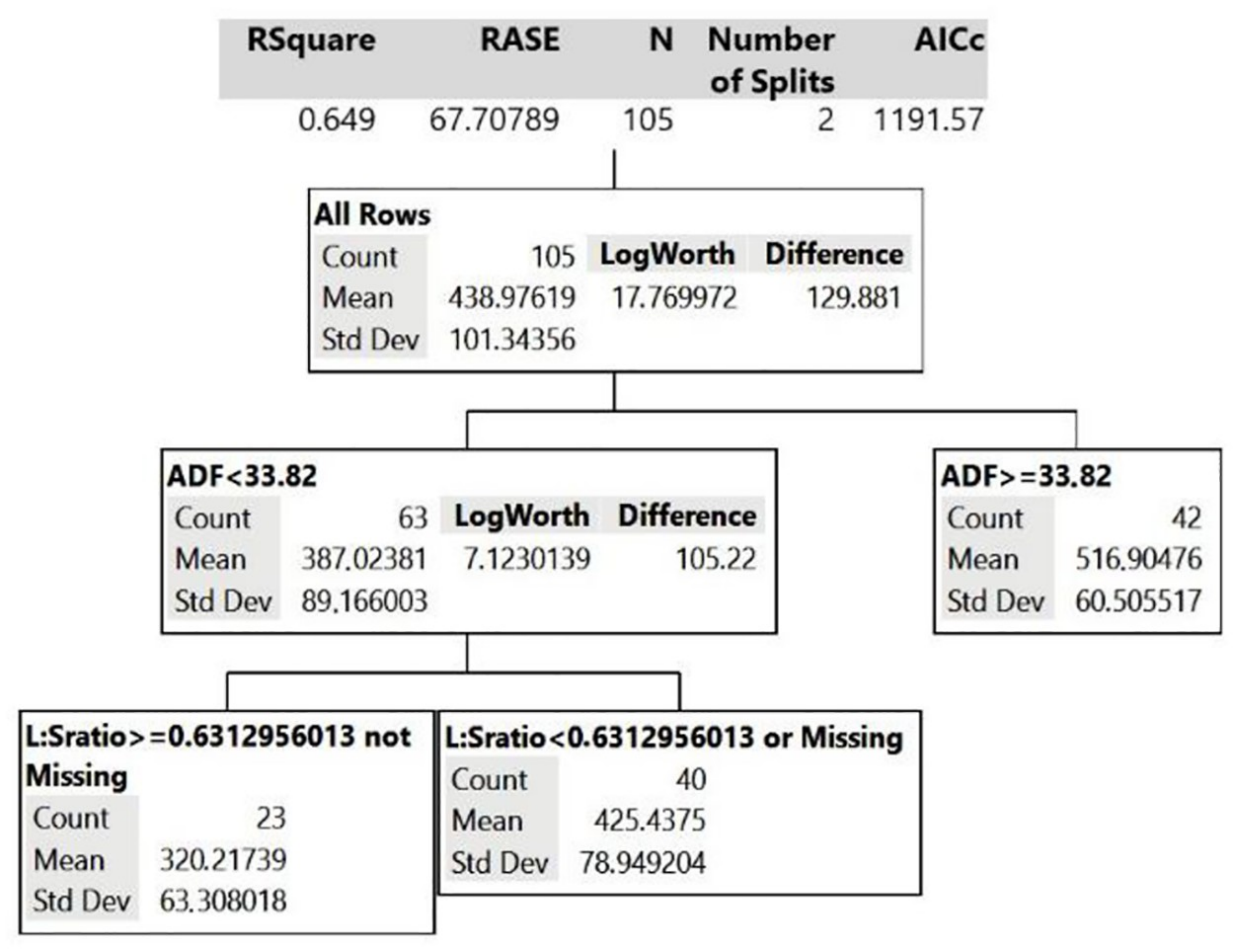
Decision Tree model for grazing time behavior variable of the animal grazing guinea grass + pigeon pea (GP canopy).

For the GP canopy, the Decision Tree (Fig 3) showed that ADF content was the most important characteristic that determines grazing time, explaining 40% of the model. The mixed system of tropical upright grass and legume with ADF below 34% of DM allows an estimated grazing time 387 minutes/day, whereas when ADF content is >34% of DM, the estimated grazing time increases more than 25% (517 minutes/day). The second branch of the Decision Tree shows that, in addition to ADF, GP leaf:stem ratio can also explain the time that lambs spend grazing. In pastures with ADF content <34%, if the leaf:stem ratio is less than 0.63, grazing time goes from 320 minutes to 425 minutes/day, a 33% increase.

## Discussion

We hypothesized that variable upright tropical pastures would affect ingestive behavior parameters of young lambs, as grasses and legumes present different structures and chemical compositions. Although, in our experiment, lambs had similar ingestive behavior in GG, PP and GP canopies, when the data are analyzed over time, we observed an interaction among the different canopies and the experimental periods. These results indicated that changes in ingestive behavior occurred and these were related to pasture morphological and histological and, consequently, nutritive values. Grazing time increased by 40% as experimental periods progressed. This change in grazing activity can be explained by the space-time variability that tropical pastures experience with advancing production cycle, since pasture maturity influences forage structural characteristics and quality, with a decrease in new leaves and a concurrent increase in stem proportion of aboveground biomass [36–38]. Under these conditions, herbivores express behavioral adaptations to maintain the ingestion of a high-quality diet. Increasing grazing time required to maintain selectivity can be an animal strategy to compensate for a reduction in grazing consumption in each bite [2, 22, 39]. High selectivity of lambs in searching for more nutritious plant parts such as leaves [40], may also require animals to graze longer because the proportion of leaves in canopies decreases over time (Table 2).

The decrease in forage quality with advancing stage of pasture maturity is well documented when analyzing the entire forage structure [36, 41–44]. Our data show that CP content did not change over time and energy values, represented by the TDN, but varied with canopy. Although there was a decrease of TDN in PP, it increased from the second to the third period. In contrast, GG and GP showed a decrease in TDN content over time. The values found in this study are related to the nutritive value of the forage consumed by the animals and not by the entire plant structure, even if forage samples were collected by grazing simulation technique (“hand plucking”) that avoids including stems in the sample. This indicates that a decrease in nutritive value, with the maturation of the pasture as a whole, is not synonymous with lower quality of diet ingested by the animals. If lambs have the opportunity, they select the most nutritious plant parts, even with the low leaf:stem ratio observed in this study.

The quality and quantity of forage mass available to the animals in this study did not limit lamb ADG to <100 g/day [45–48]. The ADGs in this trial were below expectations. According to the forage mass and chemical composition offered to the animals in this study, NRC [48] estimates that lambs should achieve gains close to or greater than 100 g/day. These low ADGs were also observed with grazing lambs on guinea grass [14], the authors suggest that this low performance is a consequence of the structure of tropical pasture, which can show high growth in a short period of time, which can affect the intake of animals. However, lambs grazing the tropical legume in our study with high productivity and nutritive value also showed lower-than-expected ADG. Lambs grazing exclusively legume pasture experienced a negative gain in the second experimental period. Our results indicate that the legume structure had a strong influence on low lamb performance once there were an increase of the legume height and a decrease of the leaf:stem ratio (r = -0.60, *P* < 0.0001). There was a correlation between leaf:stem ratio and ADG (r = 0.42, *P* < 0.0001). These results were also verified by another study [49] that suggested that, in tropical pastures, low animal performance is related to structural characteristics. The shrubby PP growth habit differs from forage legumes used in temperate pastures (*Trifolium* spp., *Medicago* spp.*; Vicia* spp., etc.). The characteristic PP woody stem, fast maturation rate and tall growth habit may make of this species a challenge for young lambs to graze.

Animals in the field use different strategies to increase or maintain consumption during grazing [2]. They can vary the mass and the frequency of the bite as well as grazing time. In our study, the increase in grazing time of the animals over the experimental period was not accompanied by a reduction in biting rate as described elsewhere [10]. Other study [10], when evaluating an upright tropical grass, found that, when animals graze large leaves, they reduce bites per minute and graze for longer periods to achieve ideal leaf capture, manipulation, chewing and swallowing. In our study, we observed an opposite behaviour. Animals in the first period had less grazing time and lower bite rate but in the last period, the animals grazed for longer and obtained the greatest bite rates. Our results may be related to bite mass because, at the beginning of the study, there was a greater density of leaves in the grazing canopy (greater leaf:stem, Table 2). This plant structure allowed lambs to achieve greater bite mass and, consequently, lower number of bites per minute. However, when leaf availability was more restricted and there was an increase in stem proportion, as happened in the second and third periods, bite mass was probably reduced and longer grazing time was necessary to maintain forage DM intake [15, 50, 51].

Rumination and idling times were relatively low in our experiment. Most ruminants spend more than 50% of the day resting and ruminating [52]. Another study [53] reported a rumination time of 466 minutes/day for lambs on millet pasture while our data showed an average of 120 minutes/12 hours of observation. However, rumination time was different among periods. In the last period of the experiment when plants were most mature, due to the greater time spent by the animals in the grazing activity, a reduction in rumination time occurred relative to that observed at the beginning of the study. Ruminating time may therefore be determined by the compensation between grazing and ruminating time during the day. Nevertheless, rumination time is also influenced by the nature of the diet and can be proportional to the cell wall content of forage [19]. In our study, there was no correlation between rumination time and forage cell wall content. We demonstrated that the time lambs spend ruminating in tropical upright pasture does not depend only on pasture chemical characteristics, but also on pasture structure and other grazing activities. There is a trade off in time among grazing activities that makes grazing time a complex factor to be predicted [39]. Because of that, we carried out a Decision Tree multivariate analysis to determine the major factors that affect grazing time.

Multivariate analysis (Decision Tree) indicated that both physical and biochemical canopy characteristics affect lamb grazing time. This result shows the major factors that affect a complex of drivers that determine lamb grazing time on tropical pasture. The vast majority of studies on animal behavior in pastoral systems report that grazing time is primarily related to the pasture structure, but there are studies that demonstrate that grazing time can be related to pasture nutritional characteristics, mostly as a response of post-ingestive consequences influenced by previous feeding experiences [54].

The Decision Tree analysis showed that leaf:stem ratio is one of the main variables, related to the structure of tropical pastures, that influences grazing time in the GG monoculture and GP mixture. This result corroborates other studies [55, 56]. These authors comment that the way leaves are presented to the animals, and the degree to which grazing adaptations can avoid stems and less digestible dead materials, is of great importance in C4 pastures. The Decision Tree for GG estimated that a leaf:stem ratio >0.37 results in a 28% reduction in the grazing time of the animals when compared to a leaf:stem ratio <0.37. This reduction in grazing time was also reported in southern Brazil with *P*. *maximum* and *Brachiaria* spp. where grazing time decreased with increasing percentage of green leaves (r = -0.63 to -0.70) [57].

In addition to greater proportion of leaves allowing easier forage intake with better nutritive value, this characteristic becomes even more important with young grazing animals, as it allows a reduction in the time and energy spent on this activity. Reducing grazing time is directly related to the reduction of energy expenditure [58, 59], so animals can redirect energy for body development. Various physiological processes contribute to this relationship, such as the work of skeletal muscle for locomotion and the use of energy by the gastrointestinal tract and liver [60–62].

Another result that shows the usefulness of the Decision Tree analysis was the effect of forage ADF content on grazing time. Although fiber content of forage is usually related to rumination time [19, 63], there is a lack of knowledge relating the chemical characteristics of forages with grazing time. Therefore, this is a novel result in describing ingestive behavior in tropical pastures. The ADF content showed a positive correlation with grazing time (r = 0.55, *P* < 0.0001). The higher the fiber content of the pasture, the greater the time spent by the animals grazing. As plant cell wall content increases, the proportion of leaves decrease, and the time spent by the animals to select more nutritious parts of the plants increases. The increase of grazing time is also associated to the need of the animals to maintain total DM intake during the day [39]. Bighorn sheep also show a negative relationship between grazing time and forage ADF content [64, 65], explained by ruminant behaviors to optimize nutrient extraction. As forage ADF content increases, the number of chews per bolus rises, increasing the manipulation for swallowing process and, consequently the time animals spend grazing.

A recent review [66] emphasizes that studies with tropical legumes are scarce regarding physical and chemical composition, and that these parameters are not considered as explanatory variables for animal responses. Therefore, the data from our study provided a clear relationship to the height of a shrubby tropical legume grazed by lambs. In particular, PP pastures, with a height of <76 cm, may allow animals to spend less time grazing compared to when it is taller. Adjusting the legume pasture height, and indirectly its chemical composition (TDN and ADF), much as with grass canopy, influences lamb grazing time. Tropical legumes have been used as an important source of protein in the production of ruminants [66, 67]; however, ideal sward management for lamb production on tropical pastures needs to be refined. Little is known about the correct way to manage tropical legumes to maximize sheep production in pastoral systems [67]. Our study indicated that grazing height of shrubby tropical legume, in our case PP, is an important factor for sheep grazing tropical pastures.

The Decision Tree analysis allowed us to visualize that, in tropical pastures composed of upright forage species that show rapid structural changes in a short period of time, it is possible to estimate the variation of sheep grazing time through relationships between forage plant physical and chemical pasture characteristics. Thus, our study was one of the first to report the influence of chemical and structural characteristics of tropical upright pastures on the time spent by lamb grazing animals, results that should guide future ruminant grazing management studies as well as practice.

## Conclusion

Our research showed that the behavior of grazing lambs in tropical upright pastures is variable, as there is a change in the grazing behavior over time, and it is related to the structural and chemical modification of these pastures. Grazing time of these animals is strongly influence by structural (leaf:stem ratio and height) and chemical (ADF, TDN) characteristics of the pasture plants. Leaf:stem ratios in tropical pastures most influenced lamb grazing time in upright perennial grass monoculture or in a mixed grass and legume canopies, while, in shrubby legume monocultures, response in grazing time was directly related to pasture height. As such, management strategies that minimize shrubby legume height and stimulate leaf regrowth and abundance compared to stems in both shrubby legumes and perennial bunchgrasses should enhance lamb performance on tropical pastures.
